# RNA sequencing-based profiling of differentially expressed microRNAs in endothelial cells from offspring of hypertensive pregnancies: a preliminary study

**DOI:** 10.3389/fmolb.2025.1520101

**Published:** 2025-07-21

**Authors:** Nurul Iffah Mohd Isa, Aslah Nabilah Abdull Sukor, Saiful Effendi Syafruddin, Mohd Faizal Ahmad, Shahidee Zainal Abidin, Mohd Helmy Mokhtar, Azizah Ugusman, Adila A. Hamid

**Affiliations:** ^1^ Department of Physiology, Faculty of Medicine, Universiti Kebangsaan Malaysia, Kuala Lumpur, Malaysia; ^2^ Faculty of Medicine, Universiti Teknologi Mara, Sungai Buloh Campus, Jalan Hospital, Sungai Buloh, Selangor, Malaysia; ^3^ UKM Medical Molecular Biology Institute, Universiti Kebangsaan Malaysia, Kuala Lumpur, Malaysia; ^4^ Department of Obstetrics and Gynaecology, Faculty of Medicine, Universiti Kebangsaan Malaysia, Kuala Lumpur, Malaysia; ^5^ Faculty of Science and Marine Environment, University Malaysia Terengganu, Kuala Nerus, Terengganu, Malaysia; ^6^ Cardiovascular and Pulmonary (CardioResp) Research Group, University Kebangsaan Malaysia, Bangi, Selangor, Malaysia

**Keywords:** endothelial dysfunction, cardiovascular disease, human umbilical vein endothelial cells, hypertensive disorders of pregnancy, microRNA, RNA sequencing

## Abstract

Offspring of mothers with hypertensive disorders of pregnancy (HDP) are at increased risk of developing endothelial dysfunction and cardiovascular disease (CVD) in adulthood. MicroRNAs (miRNAs), as key regulators of endothelial cells, may contribute to the early onset of endothelial dysfunction. However, there are limited studies characterizing the miRNA profile of endothelial cells in offspring of HDP. Therefore, this study aims to determine the miRNA expression profile of human umbilical vein endothelial cells (HUVECs) isolated from the offspring of HDP. HUVECs were obtained from both normal and hypertensive umbilical cords. RNA sequencing analysis revealed that eight miRNAs were significantly upregulated in HUVECs from HDP (p < 0.05). The target genes of these miRNAs were then predicted using four databases: miRDB, TargetScan, DIANA-microT-CDS, and miRWalk. Gene ontology, pathway enrichment, and protein-protein interaction network analyses revealed that the target genes of these miRNAs are involved in cellular functions and pathways related to angiogenesis and cellular senescence, which may contribute to endothelial dysfunction and CVD. The most significantly upregulated miRNA, hsa-miR-196a-5p expression was then validated through stem-loop RT-qPCR where its expression was significantly upregulated in hypertensive HUVEC by 6-fold as compared to normal HUVEC (p < 0.01). These findings offer insights into the role of miRNAs in the development of CVD in offspring exposed to HDP, highlighting their potential as predictive markers and therapeutic targets in the future.

## 1 Introduction

Hypertensive disorders of pregnancy (HDP) are the leading cause of morbidity and mortality in both women and newborns, accounting for up to 10% of all complications in all pregnancies (Bajpai et al., 2023). Hypertension in pregnancy can be labelled chronic if diagnosed before 20 weeks of pregnancy or *de novo* if diagnosed during or after 20 weeks of pregnancy (Brown et al., 2018). Classification of HDP includes chronic hypertension, pre-eclampsia (PE), gestational hypertension, pre-eclampsia superimposed on chronic hypertension, and white-coat hypertension as according to International Society for the Study of Hypertension in Pregnancy (ISSHP) (Magee et al., 2022). Pregnancy is often considered a ‘cardiometabolic stress test,’ where complications such as HDP may serve as early indicators of an increased risk for future cardiovascular disease (CVD). Over the past 2 decades, extensive evidence has established a strong association between HDP and CVD (Burger et al., 2022). For example, 65% of CVD events occurred in women who had a history of PE and also a higher risk of coronary heart disease, ischemic stroke and type 2 diabetes mellitus by genetic prediction (Leon et al., 2019; Rayes et al., 2023). In addition, the offspring of women with HDP are also at risk of CVD in the future. In the HUNT study conducted by Alsnes et al., blood pressure and CRP levels were found to be higher in the offspring of mothers with HDP than in the offspring of mothers with normal pregnancies (Alsnes et al., 2017). Moreover, a cohort study found that prenatal exposure of offspring to maternal PE increased the offspring’s risk of ischemic heart disease (IHD) by 33% and stroke by 34% in adolescence and young adulthood, with a higher risk of stroke in severe PE (Yang et al., 2022). Determining the factors that may contribute to the development of the disease, especially those at increased risk, is important for early prevention.

Human umbilical vein endothelial cells (HUVECs) serve as a widely used *in vitro* model for studying endothelial function and CVD. They have been extensively employed to assess endothelial dysfunction in offspring exposed to hypertensive pregnancies (Medina-Leyte et al., 2020; Reckelhoff et al., 2019). It has been shown that endothelial function in HUVECs has changed due to HDP and this change may affect the cardiovascular health of offspring exposed to HDP in the future (Brodowski et al., 2017; Yu et al., 2018). Endothelial dysfunction in HDP offspring may result from the dysregulation of microRNAs (miRNAs), which have been implicated in hypertension-associated vascular inflammation and endothelial impairment (Nemecz et al., 2016). For example, studies by Yu et al. and Ntsethe and Mackraj have found that the expression of some miRNAs, namely *miR-146a, miR-155* and *miR-222*, is associated with endothelial dysfunction in samples from women with gestational hypertension and PE (Yu et al., 2018; Ntsethe and Mackraj, 2022). *miR-146a* was found to be upregulated in HUVECs of hypertensive pregnancy and the overexpression of this miRNA decreased the vascular tube formation of HUVECs (Yu et al., 2018). Also, upregulation of miR-155 caused the suppression of migration and invasion of HUVECs through downregulation of *AKT1* gene expression (Chen et al., 2019). In addition, transfection of *miR-221/222* mimic in HUVECs reduced the wound healing and tube formation of HUVECs by decreasing c-Kit expression (Poliseno et al., 2006). These findings suggest that overexpression of these miRNAs inhibit the angiogenic activity in HUVECs, and disruption in angiogenesis is one of the key factors that cause endothelial dysfunction. Hence, dysregulation of miRNAs in HUVECs may contributed to the endothelial dysfunction in offspring of HDP.

miRNAs are small (∼22 nucleotide) non-coding RNAs that regulate gene expression post-transcriptionally by binding to the 3′-untranslated region (UTR) of target mRNAs, leading to translational repression or mRNA degradation (Matsuyama and Suzuki, 2019; Vaghf et al., 2021). Gene regulation by miRNA plays a crucial role in physiology, development and disease (Suzuki, 2023). The finding that miRNAs play a significant role in the pathogenesis of various diseases, as well as their presence and stability in body fluids, have triggered extensive research to discover their potential as non-invasive biomarkers for disease detection and prognosis (Pogribny, 2018). miRNAs have been identified as miRNAS have been identified as important regulators of many essential cellular processes, and dysregulation of miRNAs has been associated with various pathological conditions (Wronska, 2022). Previous studies showed that miRNA dysregulation also occurs in HDP (Yu et al., 2018; Munaut et al., 2016; Kim et al., 2020; Tang et al., 2019).

Further analyses of miRNAs can be performed using computational methods known as *in silico*. The prediction and characterisation of miRNA *in silico* is rapidly increasing and is more effective than laboratory-based cloning methods as it is fast and inexpensive (Chaudhary et al., 2022). Several computational databases, such as miRDB, DIANA-microT-CDS, TargetScan, and miRWalk, facilitate the prediction and characterisation of miRNA targets. These databases provide details from the literature on both *in silico* and experimental strategies related to physiological and pathological conditions (Sales and Calura, 2021). miRDB allows users to submit specific miRNA or sequences of gene targets for transcriptome-wide prediction or regulators of miRNA of any of the five species, which include humans, mice, rats, dogs and chickens; DIANA-microT-CDS uses independent models for miRNA binding in both the coding sequences (CDS) and the 3′-UTR to assess targeting in each region independently before integrating them into a single score; TargetScan allows searching for miRNA names, gene names or from poorly conserved, conserved or broadly conserved miRNA families across multiple species; miRWalk is particularly useful for performing a comprehensive assessment of miRNA binding sites from ten prediction datasets as well as for eliminating false positives (Chen and Wang, 2020; Peterson et al., 2014; Dweep et al., 2013). Integrating different algorithms in determining the miRNA targets based on the features they provide can increase the accuracy in predicting the target (Dweep et al., 2013). Understanding the role of miRNA in HDP and its impact on maternal and offspring health is not yet fully understood and remains complicated. Therefore, *in silico* analysis can help us to discover the contribution of miRNAs to disease development and serves as a precursor for further studies to understand CVD development in the offspring of women with HDP due to dysregulation of miRNAs.

## 2 Materials and methods

### 2.1 HUVEC isolation and culture

The study was approved by the Research Ethics Committee of Universiti Kebangsaan Malaysia (UKM) (Ethics Ref. No.: JEP-2023-567). Informed consent was obtained from the participants of this study, consisting of pregnant mothers with normal and hypertensive pregnancies, to participate in this study prior to the commencement of sample collection. The umbilical cords of the hypertensive group were collected among the mothers who experienced gestational hypertension, or also known as pregnancy-induced hypertension (PIH). The umbilical cords of the participants were then removed. HUVECs were isolated from the umbilical cord vein according to the method of Hamid et al. (2022). The cells from the umbilical vein wall were digested with 0.1% collagenase type I (Worthington Biochemical Corporation, Lakewood, NJ, United States). The isolated cells were then cultured in endothelial cell medium (ECM) supplemented with 1% endothelial cell growth supplement (ECGS), 5% foetal bovine serum (FBS) and 1% penicillin-streptomycin solution (ScienCell Research Laboratories, Carlsbad, CA, United States) at 37°C with 5% CO_2_ in an incubator. Cells were cultured until they reached 80% confluence and cells from passages 3 to 4 were used for downstream analysis.

### 2.2 Immunofluorescence staining of von Willebrand factor (vWF) in HUVEC

HUVECs (3 × 10^4^ cells/well) were cultured in 24-well plate (NEST, Jiangsu, China) that contained poly-L-lysine-treated 12 mm glass coverslips for 48-h or until the cells has reached 60% confluency. The cells were washed with 1× PBS (Vivantis Technologies, Selangor, Malaysia) and fixed with 4% paraformaldehyde (MERCK Sigma-Aldrich, Darmstadt, Germany) for 20 min at room temperature. Cells were then washed with 1X TBST buffer (Elabscience, Texas, United States) for three times and permeabilised with 0.1% Triton-X (MERCK Sigma-Aldrich, Darmstadt, Germany) for 15 min at room temperature. Cells were washed with 1× PBS for three times. Cells were stained according to Anti-rabbit IgG-FITC Immuno Fluorescence Staining Kit instructions (Elabscience, Texas, United States) with 0.025 mg/mL vWF polyclonal antibody (Elabscience, Texas, United States) was used as the primary antibody. The vWF expression in HUVEC was observed using Carl-Zeiss™ Axio Vert. A1 inverted microscope (Carl-Zeiss, Oberkochen, Germany).

### 2.3 Total RNA extraction

Total RNA was extracted according to the instructions of the miRNeasy Mini Kit (Qiagen, Hilden, Germany). The concentration and purity of the RNAs were determined using a spectrophotometer (DeNovix, Wilmington, DE, United States), while the integrity of the RNA was determined using an Agilent 2100 Bioanalyzer (Agilent Technologies, Santa Clara, CA, United States). The RNAs were then stored at −80°C.

### 2.4 miRNA sequencing

The differentiation of the miRNA profile in the normotensive HUVEC and the hypertensive HUVEC was performed using the RNA sequencing database, next-generation sequencing (NGS). The RNAs extracted from the samples of the two groups (normotensive and hypertensive HUVECs), where two samples from each group were submitted and processed by Novogene AIT Genomics Singapore Pte. Ltd. for library preparation and sequencing. Small RNA sequencing on Illumina Novoseq 6000 was used to generate 50 base pairs (bp) single-end reads. Raw data of fastq format were processed through custom perl and python scripts in order to obtain clean data by removing reads containing poly-N, with 5′ adapter contaminants, without 3′ adapter, containing poly A or T or G or C and low-quality reads from the raw data. Small RNA tags were then mapped to the reference sequence by Bowtie (Langmead et al., 2009) without mismatch. Since miRBase20.0 was used as a reference, the modified mirdeep2 software (Friedländer et al., 2011) and srna-tools-cli were used to obtain the potential known miRNA. miREvo (Wen et al., 2012) and mirdeep2 (Friedländer et al., 2011) were integrated to predict novel miRNA. The miRNA expression levels were estimated by TPM (transcript per million) using the normalisation formula (Zhou et al., 2010):

Normalised  expression=mapped  readcount/total  reads  x  1,000,000

Differential expression analysis of two conditions/groups was performed using the DESeq R package (1.8.3). The p-values were adjusted using the Benjamini & Hochberg method. A standard corrected p-value of 0.05 was set as the threshold for significantly different expression. The significant and highly expressed miRNAs (p-value <0.05 and fold change log_2_ ≥1.95) were selected for further analysis. Conservation status of the miRNAs was determined by using TargetScan 8.0 (https://www.targetscan.org).

### 2.5 Identification of predicted targeted genes

The predicted target genes of the eight differentially expressed miRNAs from the miRNA sequencing results were obtained from four databases, including DIANA-microT-CDS v5.0 (https://dianalab.e-ce.uth.gr/html/dianauniverse/index.php?r=microT_CDS), where the threshold for the target score was set from 0.7 to 1, TargetScan 8.0 (https://www.targetscan.org), where the genes with the positive conserved site were selected, miRDB (https://mirdb.org/), where the genes with the target score 50 to 100 were selected, and miRWalk (http://mirwalk.umm.uni-heidelberg.de/) with a binding score higher than 0.8. The settings for all databases, especially for DIANA-microT-CDS, miRDB, and miRWalk were set with moderate sensitive value as it provides good balances between sensitivity and specificity while positive conserved site for TargetScan was chosen to prevent false positive in target identification. All predicted target genes identified by these databases were analysed using Venny 2.1, a Venn diagram software (https://bioinfogp.cnb.csic.es/tools/venny/), where the genes that overlapped with four databases were selected.

### 2.6 Gene ontology (GO) and pathway enrichment analyses

The target gene candidates of the differentially expressed miRNAs were used to determine the function of the genes *via* GO and also the pathways in which the genes are involved *via* Kyoto Encyclopaedia of Genes and Genomes (KEGG) Pathway Enrichment Analysis. The bioinformatics database, Database for Annotation, Visualisation and Integrated Discovery (DAVID) v6.8 was used to identify the gene functions and also the pathways involved (Sherman et al., 2022). Official Gene Symbol was used as identifier and Gene List was chosen as the list type. The functional annotation table listed the function and pathways of differentially expressed miRNAs with a threshold number of 2, an EASE score of ≥0.1 and a number of 1,000 records. EASE score is a modified Fisher’s Exact p-value, and p-value ≤0.05 was considered to be strongly enriched. For each category, the 10 most significant terms (p < 0.05) for GO and the 20 most significant pathways (p < 0.05) were selected. The graphs of the GO and pathway enrichment analyses for DAVID were created with SRplot (http://www.bioinformatics.com.cn/en) (Tang et al., 2023).

### 2.7 Protein-protein interaction (PPI) network of target genes

In addition to identifying the gene functions and the signaling pathways involved, the hub genes were also determined by the PPI network. The genes targeted by the differentially expressed miRNAs were inserted into the Cytoscape software (version 3.10.1) to generate a complete STRING network (cut-off value of 0.4, 0 maximum additional interactors with smart limiters) (Shannon, 2003; Doncheva et al., 2019). The degree of connectivity between the 20 most important hub genes in the network was then determined using CytoHubba (Chin et al., 2014). The link between the hub genes, differentially expressed miRNAs, and related pathways and GO were visualized using ClueGo v.2.5.10 with medium specificity and 0.5 kappa score for network connectivity (Bindea et al., 2009). The p-value was set at 0.05 and corrected with Benjamini–Hochberg method. The hub genes were then further analysed with miEAA 2.0 *via* miRTargetLink 2.0 to determine miRNA-target genes over-representation enrichment analysis, with only strong validated miRNA targets were chosen (Kern et al., 2021).

### 2.8 Stem-loop reverse transcriptase quantitative polymerase chain reaction (RT-qPCR) for miRNA expression

The expression of *miR-196a-5p*, the most significantly upregulated miRNA in HUVEC exposed to HDP was further validated by using stem-loop RT-qPCR. cDNA was synthesized from 10 ng of total RNA using MMLV Reverse Transcriptase 1st-Strand cDNA Synthesis Kit (Biosearch Technologies, Hoddesdon, United Kingdom) together with the addition of 0.1 µM of stem-loop primer. The expression of *miR-196a-5p* was then detected through qPCR by using 2× miRCURY LNA miRNA SYBR Green PCR kit (Qiagen, Hilden, Germany) with additional of 0.2 µM forward and reverse primers and 1 µL of 60x diluted cDNA in 10 µL of total reaction. Primer sequences are listed in Table 1 qPCR reaction was done by using Bio-Rad CFX96™ Real-Time PCR Detection System (Bio-Rad, Hercules, CA, United States). The following qPCR protocol was used: initial denaturation at 95°C for 10 min, followed by 45 cycles of amplification at 95°C for 10 s, annealing at 59.2° for 30 s, elongation at 72°C for 10 s, and additional elongation at 40°C for 1 s. The obtained data was analyzed by using CT value through the software provided by the machine. Relative expression of hsa-miR-196a-5p was then calculated using the 2^−ΔΔCT^ method, where ∆∆CT = CT of miR-196a-5p–CT of U6. U6 gene was used as the housekeeping gene.

**TABLE 1:** Primer sequences used for qPCR.

Type of primer (5′ to 3′)	Sequence	Product size (bp)
hsa-miR-196a-5p (Forward)	CGGCTAGGTAGTTTCATGTT	93
hsa-miR-196a-5p (Reverse)	GTAGGATGCCGCTCTCAG	
U6 (Forward)	CTCGCTTCGGCAGCACA	94
U6 (Reverse)	AACGCTTCACGAATTTGCGT	
Stem-loop of hsa-miR-196a-5p	GTTGGCTCTGGTAGGATGCCGCTCTCAGGGCATCCTACCAGAGCCAAACCCCAAC	

### 2.9 Statistical analysis

The RT-qPCR data were analyzed by using GraphPad Prism 9.0 software where results were expressed in mean ± SEM. Statistical evaluation between the groups was analyzed using independent t-test and p-value <0.05 was considered statistically significant.

## 3 Results

### 3.1 Validation of vWF expression in isolated HUVEC through immunofluorescence staining

**FIGURE 1:**
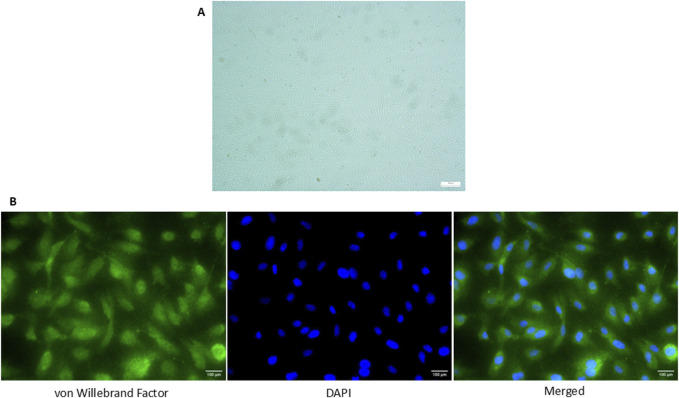
Expression of vWF in HUVEC isolated from umbilical cord *via* immunofluorescence staining. **(A)** Cobblestone morphology of the cells was seen under the inverted microscopy (×100 magnification) **(B)** Positive expression of vWF in HUVECs was detected and the nucleus of the cells was stained with DAPI (×400 magnification). The scale bar of the pictures was set at 100 µm.

All of the cells positively expressed vWF, a marker commonly used for HUVEC under the fluorescence microscopy and exhibited cobblestone morphology under inverted microscopy (Figure 1).

### 3.2 Differentially expressed miRNAs discovered in HUVEC exposed to PIH through miRNA sequencing

**FIGURE 2:**
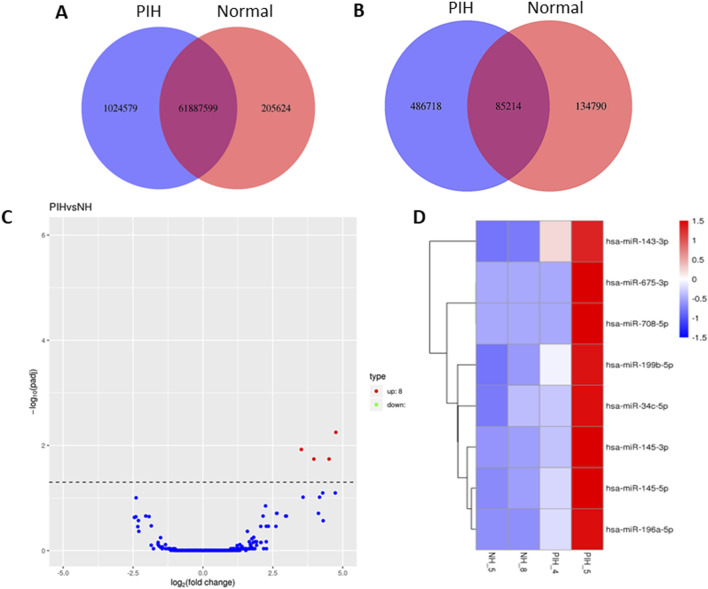
Venn diagrams of **(A)** known and **(B)** unique miRNAs identified in normal and PIH-HUVECs. **(C)** A volcano plot showing the miRNA expression profile in HUVECs, with the red dots representing significantly upregulated miRNAs. miRNAs that do not show significant expression changes are shown as blue dots. **(D)** Hierarchical clustering and heatmaps of miRNA expression profiles. The samples are arranged vertically and clustered using colour bars between the dendrogram and the heatmap. The colour key represents the relative expression of miRNAs in all samples. Red stands for higher expression levels, blue for lower expression levels.

miRNA sequencing revealed significant differences in miRNA expression between normal and PIH HUVECs. A total of 61, 887, 599 known miRNAs and 85,214 novel miRNAs were detected. Eight miRNAs were significantly upregulated in PIH samples compared to normal HUVECs, with hsa-*miR-196a-5p* was the most significant miRNA that shown large fold change, suggesting its potential role in endothelial dysfunction associated with PIH (Figure 2; Table 2.) (Details in Supplementary Material S1).

**TABLE 2:** Differentially expressed miRNAs in normal vs hypertensive HUVEC (p < 0.05).

miRNA	Log_2_ fold change	p value	Type of regulation	Conservation status
hsa-miR-196a-5p	9.6017	1.64E-09	Upregulated	Broadly conserved
hsa-miR-199b-5p	5.8767	1.77E-07	Upregulated	Broadly conserved
hsa-miR-675-3p	23.205	1.24E-06	Upregulated	Poorly conserved
hsa-miR-708-5p	7.2509	3.25E-05	Upregulated	Conserved
hsa-miR-34c-5p	4.7556	5.09E-05	Upregulated	Broadly conserved
hsa-miR-143-3p	3.5212	0.00012922	Upregulated	Broadly conserved
hsa-miR-145-3p	4.5125	0.00026234	Upregulated	Conserved
hsa-miR-145-5p	3.9692	0.00025665	Upregulated	Broadly conserved

### 3.3 Target gene prediction of differentially expressed miRNAs

**FIGURE 3:**
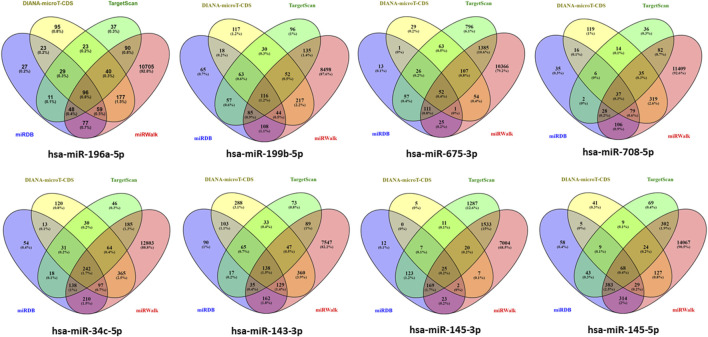
Determination of target gene numbers illustrated by Venn diagram which obtained from four databases including DIANA-microT-CDS, TargetScan, miRDB, and miRWalk for all differentially expressed miRNAs.

Genes targeted by differentially expressed miRNAs from the four databases, which are miRDB, DIANA-microT-CDS, TargetScan, and miRWalk in the Figure 3 shown total of genes targeted by hsa-*miR-196a-5p*, hsa-*miR-199b-5p*, hsa-*miR-675-3p*, hsa-*miR-708-5p*, hsa-*miR-34c-5p*, hsa-*miR-143-3p*, hsa-*miR-145-3p*, and hsa-*miR-145-5p* are 96 genes, 116 genes, 52 genes, 37 genes, 242 genes, 136 genes, 25 genes, and 68 genes, respectively. In total, 774 genes are targeted by the eight differentially expressed miRNAs with hsa-*miR-34c-5p* targeting the highest number of genes, 242 genes (Details in Supplementary Material S2).

### 3.4 GO enrichment analysis of differentially expressed genes

**FIGURE 4:**
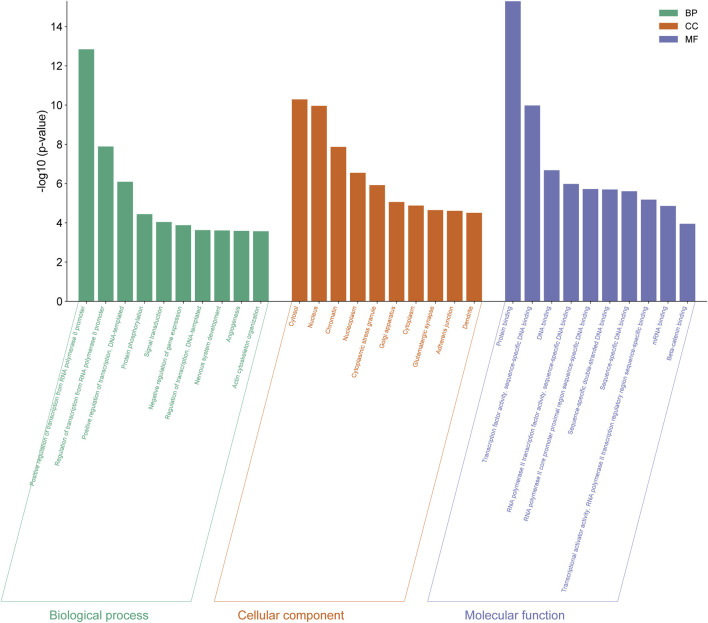
GO functional analysis of differentially expressed genes from eight differentially expressed miRNAs in normal vs PIH groups where GO were classified into three categories including biological process (BP), cellular components (CC), and molecular function (MF).

The genes expressed by all differentially expressed miRNAs were annotated by GO analysis and their functions were categorized into three categories: biological process (BP), cellular components (CC) and molecular function (MF). The top 10 functions from each category were selected based on the number of genes and the importance of the function (Figure 4) (Details in Supplementary Material S3).

The 10 enriched GO terms of biological process include positive regulation of transcription by RNA polymerase II promoter (GO:0045944), regulation of transcription by RNA polymerase II promoter (GO:0006357), positive regulation of transcription, DNA-templated (GO:0045893), protein phosphorylation (GO:0006468), signal transduction (GO:0007165), negative regulation of gene expression (GO:0010629), regulation of transcription, DNA-templated (GO:0006355), development of the nervous system (GO:0007399), angiogenesis (GO:0001525) and organisation of the actin cytoskeleton (GO:0030036).

The 10 cellular compartment GO terms include cytosol (GO:0005829), nucleus (GO:0005634), chromatin (GO:0000785), nucleoplasm (GO:0005654), cytoplasmic stress granules (GO:0010494), Golgi apparatus (GO:0005794), cytoplasm (GO:0005737), glutamatergic synapse (GO:0098978), adherens junction (GO:0005912) and dendrite (GO:0030425).

GO terms in molecular function consist of protein binding (GO:0005515), transcription factor activity, sequence-specific DNA binding (GO:0003700), DNA binding (GO:0003677), RNA polymerase II transcription factor activity, sequence-specific DNA binding (GO:0000981), RNA polymerase II core promoter proximal region sequence-specific DNA binding (GO:0000978), sequence-specific double-stranded DNA binding (GO:1990837), sequence-specific DNA binding (GO:0043565), transcription activator activity, sequence-specific binding of RNA polymerase II transcriptional regulatory region (GO:0001228), mRNA binding (GO:0003729) and beta-catenin binding (GO:0008013).

Among these significant terms, angiogenesis plays a significant role in the development of HDP, even CVD in offspring exposed to HDP as dysregulation of this process may lead to impairment of vascular function.

### 3.5 Pathway enrichment analysis of differentially expressed genes

**FIGURE 5:**
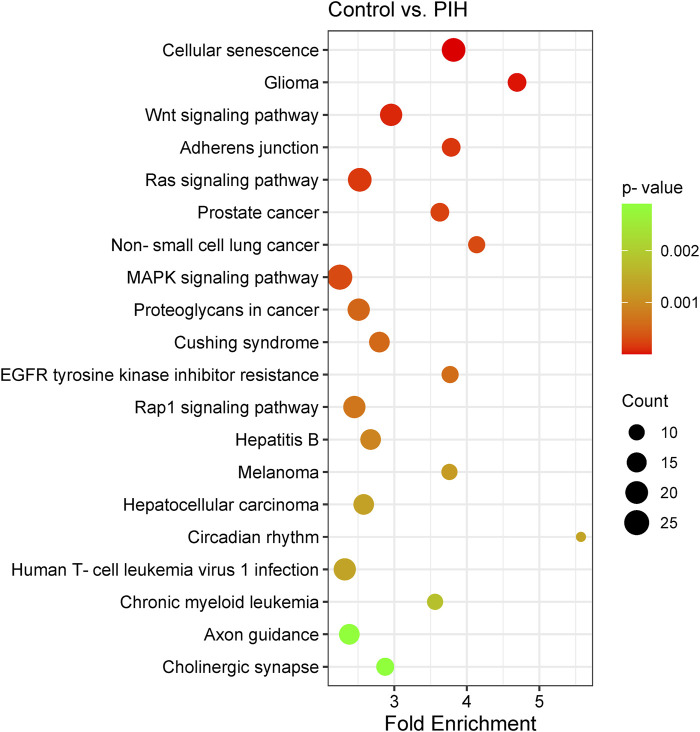
Scatter plot showing the differentially expressed genes by KEGG pathway analysis. The size of the dot represents the number of genes involved in the pathway and the range of the p-value is determined by the colour of the dot. The degree of enrichment is determined by the fold enrichment and the significance of the enrichment is determined by the p-value. A higher level of enrichment indicates a higher degree of enrichment, while a lower p-value indicates a higher significance of enrichment.

Analysis of the KEGG pathways was able to identify the related pathways involved in HDP based on the genes expressed by the eight differentially expressed miRNAs, where cellular senescence was identified to be the most significant pathway involved by the genes targeted by the differentially expressed miRNAs. The significant pathways include cellular senescence, glioma, Wnt signalling pathway, adherens junction, Ras signaling pathway, prostate cancer, non-small cell lung cancer, MAPK signaling pathway, proteoglycans in cancer, Cushing’s syndrome, EGFR tyrosine kinase inhibitor resistance, Rap1 signaling pathway, hepatitis B, melanoma, hepatocellular carcinoma, circadian rhythm, human T-cell leukaemia virus 1 infection, chronic myeloid leukaemia, axon guidance and cholinergic synapse, with p-values ranging from 2.38 × 10^−7^ to 2.90 × 10^−3^ (Figure 5) (Details in Supplementary Material S4). The finding of cellular senescence as the most significant pathway in KEGG pathway analysis may indicates that this pathway could be linked to endothelial dysfunction and risk of CVD development in offspring of HDP in the future.

### 3.6 Protein-protein interaction (PPI) network of genes targeted by differentially expressed miRNAs

**FIGURE 6:**
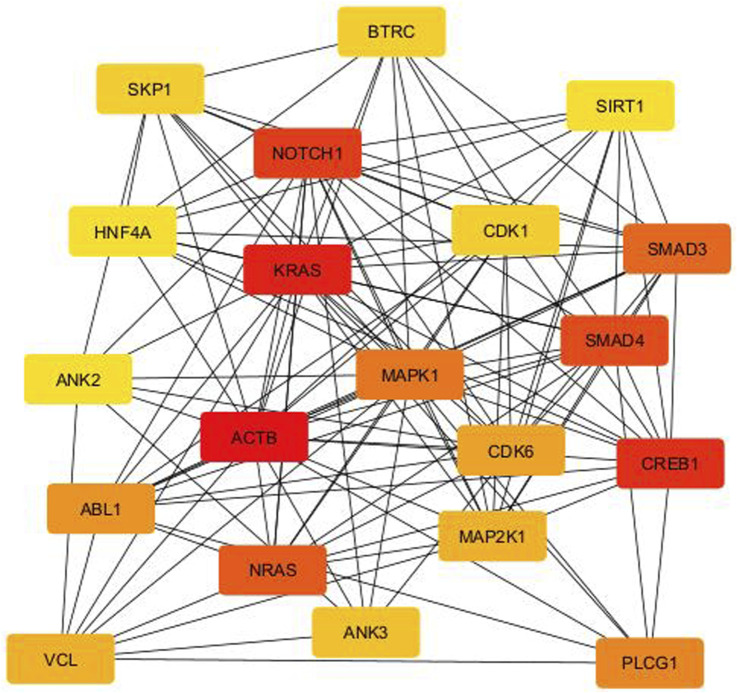
PPI network between the top 20 target genes expressed by the differentially expressed miRNAs illustrated *via* CytoHubba in CytoScape. Colour intensity of the nodes from red to yellow indicates for ranking of the nodes by degree ranking method (red to yellow–high to low).

**FIGURE 7:**
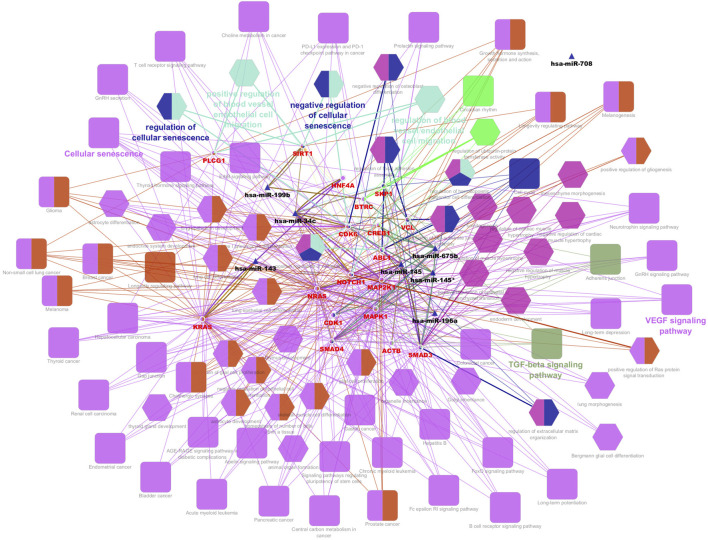
Interaction network of hub genes with the differentially expressed miRNAs, biological process (BP) in GO and pathways using ClueGO. The colour of the nodes represents for the cluster of GO and pathway. Triangle node represents for miRNA, ellipse node represents for the genes, square node represents for pathway, and hexagon node represents for BP in GO. All of the pathways and functions depicted in the figure are statistically significant (p < 0.05).

From the PPI network of eight differentially expressed miRNAs, the β-actin (ACTB) gene was found to be the nodal gene that has a high level of interaction with other genes, 98 level of interactions followed by the GTPase KRas (KRAS), cyclic AMP-responsive element-binding protein 1 (CREB1), neurogenic locus notch homolog protein 1 (NOTCH1), mothers against decapentaplegic homolog 4 (SMAD4), GTPase NRas (NRAS), mothers against decapentaplegic homolog 3 (SMAD3), mitogen-activated protein kinase 1 (MAPK1), 1-phosphatidylinositol 4,5-bisphosphate phosphodiesterase gamma-1 (PLCG1), tyrosine-protein kinase Abelson murine leukaemia viral oncogene homolog 1 (ABL1), cyclin-dependent kinase 6 (CDK6), mitogen-activated protein kinase 1 (MAP2K1), vinculin (VCL), ankyrin 3 (ANK3), cyclin-dependent kinase 1 (CDK1), beta-transducin repeat-containing (BTRC), S-phase kinase-associated protein 1 (SKP1), NAD-dependent protein deacetylase sirtuin-1 (SIRT1), hepatocyte nuclear factor 4 alpha (HNF4A) and finally, ankyrin 2 (ANK2) (Figure 6) (Details in Supplementary Material S5). The results of the degree of connectivity for the 20 most important genes are listed in Table 3. Enrichment analysis on the hub genes by ClueGO also supported the enrichment data of the overall genes done through the DAVID Bioinformatic Database where cellular senescence was among the most significant pathways targeted by the genes together with few pathways that are related to angiogenesis, including TGF-beta and VEGF signaling pathways (Figure 7) (Details in Supplementary Material S6). This shown that the hub genes are most probably play significant role in regulating cellular senescence and angiogenesis in HUVEC of HDP with their respective target miRNAs. In addition, the miRNA-target genes over-representation enrichment analysis further supported the interaction in the PPI network where SIRT1 targeted by hsa-*miR-199b-5p* was the most significant gene to be targeted by a cluster of miRNAs (Table 4). As the most significantly enriched gene with high degree of connectivity, dysregulation of SIRT1 expression might be significant in HUVEC exposed to HDP. Overall, based on the enrichment analyses, most of the hub genes are targeted by the differentially expressed miRNAs except for hsa-*miR-708-5p*, and most of the hub genes and miRNAs were found to be associated to pathways which are related to endothelial dysfunction (Table 5).

**TABLE 3:** Top 20 genes targeted by the miRNAs ranked by degree ranking method and score of the connectivity degree in the PPI network between the genes.

Rank	Gene	Degree score
1	ACTB	98
2	KRAS	79
3	CREB1	55
4	NOTCH1	52
5	SMAD4	50
6	NRAS	48
7	SMAD3	47
8	MAPK1	44
9	PLCG1	42
10	ABL1	39
11	CDK6	38
12	MAP2K1	36
12	VCL	36
14	ANK3	35
15	CDK1	34
15	BTRC	34
15	SKP1	34
18	SIRT1	33
18	HNF4A	33
18	ANK2	33

**TABLE 4:** miRNA-hub genes over-representation enrichment analysis with strongly validated miRNA targets.

Genes	Enrichment	P-value	P-adjusted	Q-value	Related miRNAs/precursors
SIRT1	over-represented	3.49E-29	2.26E-25	2.26E-25	hsa-miR-199b-5p
NOTCH1	over-represented	4.01E-27	1.73E-23	1.73E-23	hsa-miR-34c-5p
SMAD3	over-represented	4.25E-24	1.37E-20	1.37E-20	hsa-miR-145-3p; hsa-miR-145-5p; hsa-miR-708-5p
SMAD4	over-represented	7.20E-23	1.55E-19	1.55E-19	hsa-miR-145-5p; hsa-miR-34c-5p
CDK6	over-represented	1.46E-22	2.70E-19	2.70E-19	hsa-miR-145-5p; hsa-miR-34c-5p
KRAS	over-represented	2.74E-22	4.44E-19	4.44E-19	hsa-miR-143-3p
CREB1	over-represented	3.93E-10	6.20E-08	6.20E-08	
HNF4A	over-represented	9.42E-09	8.13E-07	8.13E-07	hsa-miR-34c-5p
MAP2K1	over-represented	3.26E-08	2.17E-06	2.17E-06	hsa-miR-34c-5p
MAPK1	over-represented	7.12E-08	4.01E-06	4.01E-06	hsa-miR-196a-5p; hsa-miR-143-3p
NRAS	over-represented	9.66E-08	5.08E-06	5.08E-06	hsa-miR-145-5p
CDK1	over-represented	3.14E-07	1.33E-05	1.33E-05	
ACTB	over-represented	6.49E-06	0.000145	0.000145	hsa-miR-145-5p; hsa-miR-196a-5p
BTRC	over-represented	1.61E-05	0.00029	0.00029	
VCL	over-represented	0.000195	0.002085	0.002085	hsa-miR-196a-5p
ANK2	over-represented	0.000947	0.006271	0.006271	
PLCG1	over-represented	0.00218	0.012914	0.012914	

**TABLE 5:** Summary of miRNAs association with the target genes and related pathways in endothelial dysfunction.

miRNAs	Number of predict targets	Targeted hub genes	Involvement in key pathways
hsa-miR-196a-5p	96	ACTB, MAPK1, VCL, ABL1, NRAS	VEGF signaling pathway, ErbB signaling pathway
hsa-miR-199b-5p	116	SIRT1, BTRC, KRAS1	Cellular senescence
hsa-miR-675-3p	52	SKP1, BTRC, CREB1, CDK1, CDK6, MAPK1, SMAD4, SMAD3	VEGF signaling pathway, TGF-beta signaling pathway, cellular senescence
hsa-miR-708-5p	37	SMAD3	
hsa-miR-34c-5p	242	MAP2K1, CDK6, NOTCH1, HNF4A, SIRT1, SMAD4, PLCG1, VCL	Cellular senescence, circadian rhythm
hsa-miR-143-3p	138	SMAD3, MAPK1, KRAS, HNF4A, CDK1, SKP1	TGF-beta signaling pathway, cellular senescence
hsa-miR-145-3p	25	CREB1, CDK1, CDK6, SMAD4, MAPK1	Cellular senescence
hsa-miR-145-5p	68	SMAD4, SMAD3, CDK6, ACTB, NRAS, SKP1	Cellular senescence, cell cycle

### 3.7 Validation of *miR-196a-5p* expression in HUVEC exposed to HDP

**FIGURE 8:**
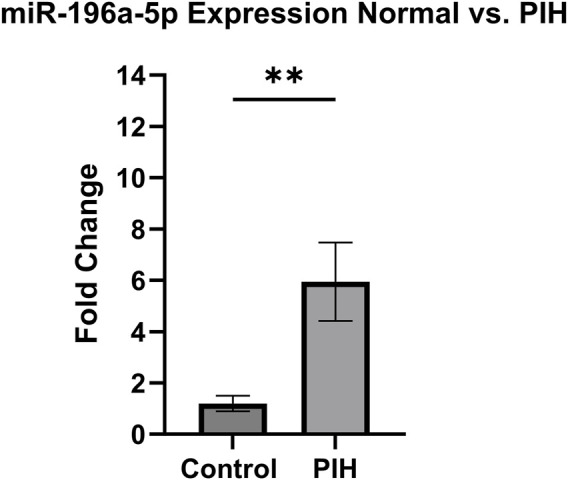
Validation of miR-196a-5p expression in normal vs PIH HUVEC *via* stem-loop RT-qPCR. Values are shown as mean ± SEM, n = 6, **p < 0.01 vs normal. Result was analyzed using Independent T-test.

Based on the miRNA sequencing, *miR-196a-5p* was discovered to be the most significantly upregulated miRNA in HUVEC exposed to HDP. Therefore, *miR-196a-5p* was selected to be validated and further quantified by using stem-loop RT-qPCR. Based on Figure 8, *miR-196a-5p* expression was significantly upregulated by 6-fold in HUVEC of HDP as compared to normal HUVEC (p < 0.01). This validation may suggest the upregulation of *miR-196a-5p* in HUVEC exposed to HDP may play a significant role in regulating genes that may lead endothelial dysfunction in offspring exposed to HDP.

## 4 Discussions

This study identified eight miRNAs that were significantly upregulated in HUVECs derived from pregnancies complicated by hypertension, suggesting a potential regulatory role in endothelial dysfunction. These miRNAs are known to influence key vascular processes, including angiogenesis, endothelial inflammation, and cellular senescence, which are critical factors in HDP and CVD risk in offspring. Notably, hsa-*miR-196a-5p* exhibited the most substantial differential expression, indicating its potential as key regulators in this pathophysiological process. Several miRNAs which were involved in HDP, especially PE, as reported in previous studies were matched with the miRNA sequencing result (Illarionov et al., 2022; Enquobahrie et al., 2011; Lv et al., 2019; Du et al., 2020). hsa-*miR-34c-5p* was upregulated in a plasma blood sample from pregnant women at high risk of preterm birth during the first trimester of pregnancy (Illarionov et al., 2022). In contrast to the current sequencing result, hsa-*miR-34c-5p* was found to be downregulated in PE (Enquobahrie et al., 2011). In addition, the expression of *miR-145-5p* decreased in PE placenta compared to control (Lv et al., 2019). Lv et al. explained that inhibition of *miR-145-5p* ex-pression in trophoblast cells decreased cell viability and invasion, while overexpression of *miR-145-5p* increased the efficacy of trophoblast cell invasion (Lv et al., 2019). Expression of *miR-199b-5p* was downregulated in PE serum (Du et al., 2020). *miR-199b-5p* may regulate the expression of caveolin 1 (CAV1), a gene that controls glomerular endothelial cell permeability associated with proteinuria in PE (Du et al., 2020). Among all of the miRNAs, hsa-*miR-196a-5p* was the most significant upregulated miRNA in HUVEC exposed to HDP, and this finding was confirmed through the validation of the miRNA expression through stem-loop RT-qPCR, where *miR-196a-5p* was upregulated in HUVEC exposed to HDP by 6-fold. Although findings on *miR-196a-5p* were mostly related to cancer research as a proto-oncogenic miRNA, there were growing evidences on the involvement of this miRNA in endothelial dysfunction as the overexpression of miR-196a-5p suppressed cell proliferation, and reduced the expression of Vascular Endothelial Growth Factor (VEGF), an angiogenesis key regulator in endothelial cells (Tong et al., 2024; Pin et al., 2012). The upregulation of *miR-196a-5p* might play a significant role in endothelial dysfunction in offspring of women with HDP.

GO showed that angiogenesis, a crucial process that plays an important role in HDP, especially in PE and also in CVD, is significantly enriched and that several of the genes targeted by the differentially expressed miRNAs are involved in this process. In PE, the level of VEGF and placental growth factor (PlGF) is reduced, while the level of soluble Flt-1 (sFlt-1) increases (Lam et al., 2005). This is due to VEGF and PlGF binding to circulating sFlt-1 instead of the target receptor, resulting in vasoconstriction and endothelial dysfunction, including increased secretion of endothelin-1, a vasoconstrictor, and a reduction in the generation of endothelial nitric oxide, a potent vasodilator (Lam et al., 2005; Karumanchi, 2016; Rana et al., 2020). The disruption of angiogenesis also can lead to impairment of endothelial function. Some of the hub genes, including NOTCH1, SMAD3, and SMAD4 were identified to be involved in angiogenesis (Li et al., 2022; Funahashi et al., 2010; Arderiu et al., 2015; Wang et al., 2023). As the expression of these genes were regulated by miRNAs identified from the miRNA sequencing, where NOTCH1 expression was regulated by hsa-*miR-34c-5p*, SMAD3 by hsa-*miR-145-3p*, hsa-*miR-145-5p* and hsa-*miR-708-5p*, and SMAD4 by hsa-*miR-145-5p* and hsa-*34c-5p* (Table 4), there were no evidences that the dysregulation of these miRNAs may influence the angiogenesis impairment in HUVEC. However, the expression of these miRNAs with their respective target genes on suppressing cell proliferation, migration, and invasion that directly reduced the angiogenesis of cancer cells by suppressing the expression of these three genes may provide insights on the dysregulation of these miRNAs that may influences angiogenesis impairment in HUVEC exposed to HDP through downregulation of NOTCH1, SMAD3, and SMAD4 (Wei et al., 2020; Huang et al., 2015; Li et al., 2017; Zhou et al., 2020).

When analysing the enrichment of metabolic pathways, cellular senescence was identified as the most important metabolic pathway targeted by the genes among the 20 most important metabolic pathways. Previous findings have shown that women with HDP, especially PE have characteristics of cells in a state of senescence, characterised by the secretion of the senescence-associated secretory phenotype (SASP). Suvakov et al. found that compared to women with normal pregnancy, the expression of SASP components, including TNF-α and MCP-1, and p16^INK4A^, a cellular senescence biomarker, are significantly higher in women with PE (Suvakov et al., 2021). In addition, women with peripartum cardiomyopathy, an unknown form of pregnancy-induced heart failure associated with PE, showed significant upregulation of SASP markers, with Activin A, a protein associated with dysregulation of cardiac function, being the highest marker expressed in the placenta of PE women (Roh et al., 2024). Women with a history of PE therefore have a higher risk of developing CVD in the future, as the findings of Suvakov et al. show that women with a history of pre-eclampsia had a worse ageing profile compared to normal pregnancies, such as increased levels of leptin/adiponectin and extracellular vesicles positive for tissue factor, as well as a decrease in urinary α-klotho levels (Suvakov et al., 2024). They were also four times more likely to develop CVD than normotensive women (Suvakov et al., 2024).

The genes SIRT1, CDK1 and SMAD3, which are among the genes with the highest degree of connectivity in the PPI, are involved in the cellular senescence pathway, and dysregulation of these genes contributes to the development of CVD. SIRT1 is a gene that is particularly highly expressed in endothelial cells of arteries, veins and capillaries and plays a crucial role in endothelial cell senescence (Hwang et al., 2022). SIRT1 is able to protect vascular endothelial cells from oxidative stress, inflammation and autophagy, and silencing of SIRT1 promotes the development of PE (Viana-Mattioli et al., 2020; Wang et al., 2022; Yu et al., 2023). In addition, inhibition of the CDK1 and SMAD3 genes has the potential to be used as a therapy for CVD, as SMAD3 knockout in mice and cardiac fibroblasts are protected from the occurrence of cardiac fibrosis and inflammation, and suppression of CDK1 alleviates vascular calcification in a diabetic mouse model by preventing the conversion of endothelial lineage cells into osteoblast-like cells (Huang et al., 2010; Zhao et al., 2024). The PPI network connectivity result was validated with miRNA-target gene over-representation analysis where almost all of the top 20 hub genes were significantly enriched with their respective miRNAs except for SKP1, ABL1 and ANK3 genes. Absence of these three genes in the enrichment analysis happened as miRNA targets were filtered by choosing only strong validated miRNA targets, as it only includes the target which was validated with RT-qPCR, Western blot, and luciferase reporter assay based on the previous findings. SIRT1 was the most significant gene targeted by a cluster of few miRNAs including hsa-*miR-199b-5p*. Previous finding revealed that SIRT1, a deacetylase, is crucial in regulating cellular senescence (Lee et al., 2023). Study by Hayakawa et al. discovered that cells in senescence state were accumulated with SASP components due to the depletion of SIRT1, and acetylation levels were increasing (Hayakawa et al., 2015). Deacetylation of SASP component in their promoter regions by SIRT1 able to suppress the induction of cell senescence (Hayakawa et al., 2015). Moreover, SIRT1 acts as a vasoprotector by promoting the production of endothelial nitric oxide synthase (eNOS), which important in maintaining relaxation of blood vessels (Wu et al., 2021; Law et al., 2024; Man et al., 2019). *miR-199b-5p* negatively regulated SIRT1 as it suppressed the expression of the gene (Shen et al., 2016; Cai et al., 2024). Thus, the upregulation of *miR-199b-5p* in HUVEC exposed to HDP based on our sequencing result may suggest possible mechanism of SIRT1 downregulation in HUVEC which induces cellular senescence in endothelium of offspring exposed to HDP and may lead to future CVD development.

The senescence of endothelial cells leads to a reduction in nitric oxide production and the ability of cells to repair themselves through angiogenesis, as well as an increase in reactive oxidative species, which leads to various dysfunctions in the cardiovascular system (Bitar, 2019; Yan et al., 2021). Production of NO is crucial for relaxation of blood vessel and reduction of NO production in the endothelial cells may lead to vascular dysfunction (Han and Kim, 2023). In addition, the offspring of women with HDP were found to have impaired endothelial and vascular functions in early life, which increases the likelihood that the offspring will develop cardiovascular disease in the future (Abdull Sukor et al., 2022). The angiogenic capabilities in HUVECs from hypertensive women are reduced as the total length of tubules and the number of branch points decrease due to the overexpression of miR-146a in the hypertensive samples (Yu et al., 2018). Moreover, a decrease in nitric oxide production was found in HUVECs from PE patients (Veas et al., 2011). The impairment of NO production due to the cellular senescence led to the reduction of VEGF which ultimately affect the angiogenic ability of the blood vessel (Ungvari et al., 2018). Impaired angiogenesis, can lead to CVD due to the decreased blood supply to tissues, potentially causing ischaemia and worsening conditions including heart failure and atherosclerosis (Ungvari et al., 2018). Evidences suggest offspring of women with HDP experienced vascular remodelling as ultrasonography revealed that foetal aortic intima-media thickness, umbilical artery pulsatility index (PI), foetal aortic PI and mean uterine artery PI were higher in foetuses with late-onset maternal gestational hypertension than in foetuses of mothers with normal blood pressure (Veas et al., 2011). These early-life changes in cardiac structure observed in PIH offspring, especially in premature babies, are associated with higher blood pressure (Lazdam et al., 2010). These evidences suggest that endothelial dysfunction may contribute to the development of CVD in the offspring of women with hypertensive pregnancies through endothelial cellular senescence and impairment of angiogenesis.

The formation of *in silico* models is supported by constantly evolving experimental and analytical methods capable of providing high-throughput biological data with a wealth of information (Barh et al., 2014). Therefore, *in silico* disease models are useful to improve our understanding of the biology of the disease, suggest new treatment approaches and provide clues for experimental design and clinical trials to explore new therapeutic options (Barh et al., 2014). Furthermore, the cost of conducting an *in silico* analysis is low and the results are readily available. However, there were also some limitations when conducting this experiment. Firstly, there were discrepancies between the sequencing result and the result of previous studies in which *miR-34c-5p*, *miR-199b-5p* and *miR-145-5p* were upregulated in samples from HUVECs exposed to hypertensive pregnancies. However, previous studies have shown that the expression of these three miRNAs was reduced in PE (Enquobahrie et al., 2011; Lv et al., 2019; Du et al., 2020). The limited number of samples used for sequencing may have contributed to the differing results, as small sample sizes increase the risk of false positives and reduce the reproducibility of the experiment. Additionally, such sample sizes may not accurately represent the larger population, as they are more prone to random variability, which affects the generalisability of the findings. The type of sample used can also influence miRNA regulation. Certain miRNAs are expressed in specific cell types or tissues, which could explain discrepancies in miRNA profiling between different sample sources (Chugh and Dittmer, 2012). For instance, previous miRNA expression analyses have used plasma, trophoblast cells, and placental tissue. Therefore, it is essential to validate all miRNAs expressed in hypertensive HUVECs to confirm their expression. Gene Set Enrichment Analysis (GSEA) can be done to complement the DAVID enrichment analysis, as GSEA is more sensitive for detecting pathway-level perturbations, especially when gene lists are small or noisy. Biological validation is also necessary to demonstrate the relationship between miRNAs, their target genes, and associated signaling pathways in the disease. This could be achieved through assays such as scratch and transwell migration tests, and the Griess assay to evaluate nitric oxide (NO) production in normal *versus* HDP HUVECs. These validations support *in silico* findings and enhance understanding of how miRNAs may contribute to cardiovascular disease development in offspring of HDP patients due to endothelial dysfunction. Furthermore, incorporating diverse cell types in future studies is recommended to better reflect the tissue’s complexity and function—something this study lacked. Besides the validation of the bioinformatic result, it is important to consider proper quality control measures were taken during cell culture practices, as its neglection may affect the validity of the results. For example, cultured cells are prone to be contaminated with mycoplasmas due to their small size and elasticity to pass-through the filter during tissue media culture sterilization process (Young et al., 2010). Because of the absence of *mycoplasma* detection in this study, it is recommended to perform *mycoplasma* detection methods which include *mycoplasma* culture on agar plate using the test sample, DNA staining with Hoescht or DAPI, and PCR-based method to detect the presence of *mycoplasma*. Other than *mycoplasma* detection, cell line authentication is essential, especially for primary cell culture. vWF and CD31 are among the known protein markers expressed by HUVEC (Medina-Leyte et al., 2020). Although the isolated cells were validated with the presence of vWF through immunofluorescence staining in this study, it is recommended to validate the cells with CD31 for further confirmation. Diagnostic tests were not conducted in the current study. However, it is recommended that future research includes receiver operating characteristic (ROC) analysis and validation using external cohorts, as microRNAs have the potential to serve as biomarkers for disease detection. Hence, an *in silico* study is useful to gain insights into the pathogenesis of a disease and to use it as a starting point for an experiment, however validation of miRNAs expression and functional assays are crucial to confirm the results obtained from the bioinformatic analysis.

## 5 Conclusion

In summary, this study has shown that eight miRNAs were differentially expressed in HUVECs exposed to hypertensive pregnancies with upregulation of hsa-miR-196a-5p, which was the most significantly upregulated miRNA expressed in hypertensive HUVEC was confirm through stem-loop RT-qPCR. Bioinformatic analyses predicted that the angiogenesis and cellular senescence signaling pathway may contribute to endothelial dysfunction in offspring exposed to HDP which may contribute to the development of CVD in the future. The results of *in silico* analysis may be useful to determine the relationship between miRNA and its role in disease development, but it is necessary to perform functional studies for validation.

## Data Availability

The original contributions presented in the study are publicly available. This data can be found here: https://www.ncbi.nlm.nih.gov/geo/query/acc.cgi?acc=GSE302001.
